# New Frontiers for Deep Brain Stimulation: Directionality, Sensing Technologies, Remote Programming, Robotic Stereotactic Assistance, Asleep Procedures, and Connectomics

**DOI:** 10.3389/fneur.2021.694747

**Published:** 2021-07-22

**Authors:** Aristide Merola, Jaysingh Singh, Kevin Reeves, Barbara Changizi, Steven Goetz, Lorenzo Rossi, Srivatsan Pallavaram, Stephen Carcieri, Noam Harel, Ammar Shaikhouni, Francesco Sammartino, Vibhor Krishna, Leo Verhagen, Brian Dalm

**Affiliations:** ^1^Department of Neurology, The Ohio State University Wexner Medical Center, Columbus, OH, United States; ^2^Department of Psychiatry, The Ohio State University Wexner Medical Center, Columbus, OH, United States; ^3^Medtronic PLC Neuromodulation, Minneapolis, MN, United States; ^4^Newronika, Milan, Italy; ^5^Abbott Laboratories, Neuromodulation Division, Austin, TX, United States; ^6^Boston Scientific Neuromodulation, Valencia, CA, United States; ^7^Center for Magnetic Resonance Research, University of Minnesota Medical School, Minneapolis, MN, United States; ^8^Department of Neurosurgery, The Ohio State University Wexner Medical Center, Columbus, OH, United States; ^9^Movement Disorder Section, Department of Neurological Sciences, Rush University, Chicago, IL, United States

**Keywords:** deep brain stimulation, robotic surgery, directionality, asleep, sensing, local field potential, connectomics, telemedicine

## Abstract

Over the last few years, while expanding its clinical indications from movement disorders to epilepsy and psychiatry, the field of deep brain stimulation (DBS) has seen significant innovations. Hardware developments have introduced directional leads to stimulate specific brain targets and sensing electrodes to determine optimal settings *via* feedback from local field potentials. In addition, variable-frequency stimulation and asynchronous high-frequency pulse trains have introduced new programming paradigms to efficiently desynchronize pathological neural circuitry and regulate dysfunctional brain networks not responsive to conventional settings. Overall, these innovations have provided clinicians with more anatomically accurate programming and closed-looped feedback to identify optimal strategies for neuromodulation. Simultaneously, software developments have simplified programming algorithms, introduced platforms for DBS remote management *via* telemedicine, and tools for estimating the volume of tissue activated within and outside the DBS targets. Finally, the surgical accuracy has improved thanks to intraoperative magnetic resonance or computerized tomography guidance, network-based imaging for DBS planning and targeting, and robotic-assisted surgery for ultra-accurate, millimetric lead placement. These technological and imaging advances have collectively optimized DBS outcomes and allowed “asleep” DBS procedures. Still, the short- and long-term outcomes of different implantable devices, surgical techniques, and asleep vs. awake procedures remain to be clarified. This expert review summarizes and critically discusses these recent innovations and their potential impact on the DBS field.

## Introduction

Multiple innovations have improved the field of deep brain stimulation (DBS) over the last decade. Here, we sought to summarize the main advancements in clinical indications, hardware development, software innovations, and surgical procedures and discuss the upcoming frontiers for DBS development in neurological and psychiatric disorders.

## Update on DBS Indications and Targets

### DBS in Movement Disorders

**Figure 1:**
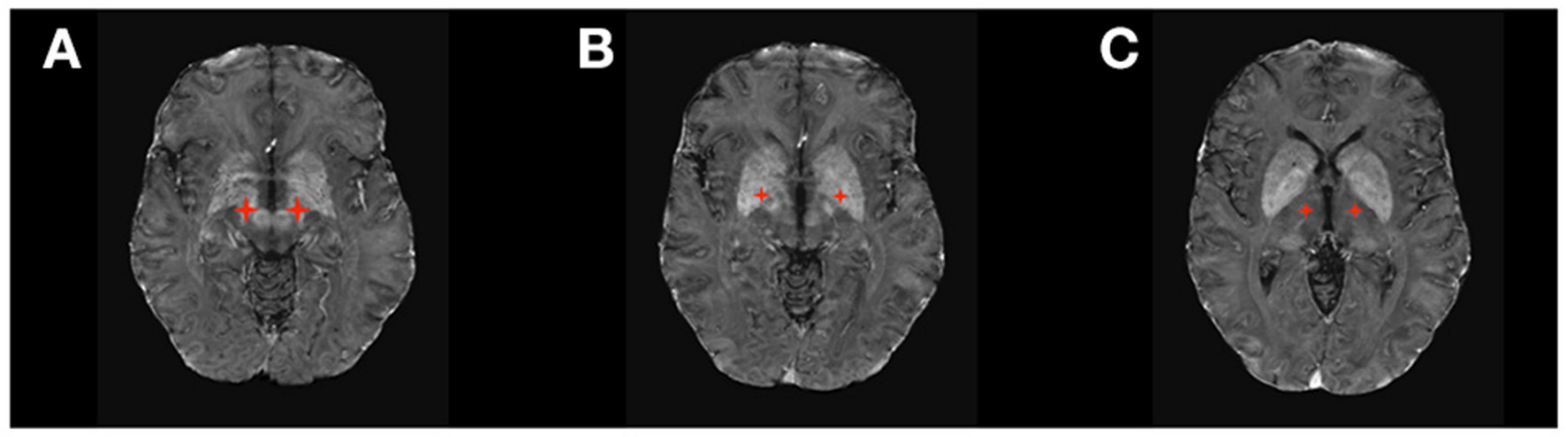
DBS targets in Movement Disorders. **(A)** Subthalamic Nucleus (STN); **(B)** Ventral Intermediate Thalamic Nucleus (VIM); **(C)** Globus Pallidus Pars Interna (GPi).

DBS is a very well-established treatment for movement disorders. With over 60,000 patients with Parkinson disease (PD), essential tremor (ET), dystonia, and Tourette syndrome implanted with DBS in the United States and more than 160,000 worldwide, DBS has demonstrated long-term efficacy on multiple aspects of movement disorders, including motor symptoms (1–5), quality of life (6), and quality-adjusted life expectancy (QALY) (7). Three main DBS targets (Figure 1) have been traditionally used in movement disorders: the subthalamic nucleus (STN), the globus pallidus pars interna (GPi), and the ventral intermediate (Vim) and ventral oralis posterior (VOP) nuclei of the thalamus.

The STN is the most common target for PD and, more recently, a target for dystonia. It has a broad range of cortical and subcortical connections (8). Cortical pathways include those connecting the posterior STN with the sensorimotor cortex, the mid-STN with the associative cortex, and the anterior STN with the limbic cortex (9). Subcortical pathways connect the STN to the caudate, putamen, pedunculopontine nucleus, globus pallidus internal and external segments (GPi/GPe), substantia nigra, substantia innominata, hypothalamus, olfactory tubercle, and mamillary bodies. Stimulation of each of these pathways affects different motor and non-motor networks. In particular, stimulation of the motor pathway results in improved PD-associated rigidity, tremor, and bradykinesia (10).

The GPi is the primary target for generalized dystonia (3), an alternative target for PD—particularly when associated with severe dyskinesia or mild cognitive impairment (2), and a possible target for Tourette syndrome (5). Tracing studies (11) showed that the GPi has projections to several motor and non-motor structures, including the premotor neurons of the ventral tier thalamic nuclei, the centro-median/parafascicular thalamic complex, and the brainstem pedunculopontine nucleus. These pallido-fugal fibers emerge either through the ansa lenticularis (AL) or the lenticular fasciculus (LF) and project to the thalamus and the brainstem through the Forel's field H.

The Vim receives fibers of the dentato-rubro-thalamic tract (DRTT), which is a white matter bundle that originates in the contra-lateral cerebellar dentate nucleus of the cerebellum, traverses to the ipsilateral superior cerebellar peduncle and then partially decussates in the midbrain to reach the thalamus, and finally terminates within the primary motor cortex. Several clinical trials demonstrated Vim-DBS efficacy in the treatment of medical-refractory ET (4) and PD (12). The VOP receives both fibers from the DRTT and the globus pallidus, making it an effective target for dystonic tremor (13).

### DBS in Epilepsy

DBS has shown positive results in patients with drug-resistant epilepsy (DRE), which accounts for up to 30% of all cases of epilepsy (14). Some patients with DRE benefit from surgery to remove the seizure focus. Still, nearly 40% of patients with DRE may not be candidates for resection surgery if their seizures originate from multiple cortical foci or their seizure focus is an eloquent cortical area that cannot be resected without unacceptable neurological deficit (15). Furthermore, approximately 50% of patients who undergo resection surgery continue to have seizures (16). In general, brain stimulation for the treatment of epilepsy can employ at least three different strategies according to the pathogenic mechanism underlying the seizure network: Responsive neurostimulation (RNS) delivers electrical stimulation directly to the epileptogenic region in response to seizure activity recorded in that area (17, 18), vagus nerve stimulation (VNS) interrupts seizure networks indirectly by delivering electrical stimulation to vagus nerve afferents in the neck, thus stimulating brainstem nuclei with wide cortical projections (19), and DBS modulates the subcortical targets connected to the cortical seizure network (17, 18, 20). As such, VNS and DBS can be used to treat focal epilepsy not amenable to resection and multifocal epilepsy.

**Figure 2:**
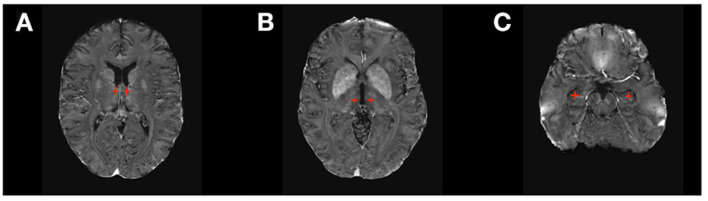
DBS targets in Epilepsy. **(A)** Anterior Thalamic Nucleus (ANT); **(B)** Centromedian Thalamic Nucleus (CM); **(C)** Hippocampus.

DBS received FDA approval in 2018 for the treatment of DRE based on evidence from the SANTE Trial (21, 22), a double-blinded randomized clinical trial of bilateral stimulation of anterior nucleus of the thalamus (ANT) for DRE (Figure 2). This study showed a 40% seizure frequency reduction in the stimulated group compared with 14.5% in the control group at 3 months. Encouragingly, DBS of the ANT, as observed in other indications, also shows progressive improvement in effect over time. In the unblinded phase of SANTE trial participants, there was 69% median seizure frequency reduction at 5 years (22). Consistent with ANT location within the medial limbic circuit, the best seizure control effect of stimulation was seen for temporal lobe epilepsy (76% improvement) compared to epilepsies of the frontal lobes (59%) or other locations (68%).

The efficacy of DBS of other brain structures to treat epilepsy remains inconclusive due to the lack of large randomized clinical trials (RCT). Stimulation of the hippocampus has been investigated as a treatment for medial temporal lobe DRE not amenable for resection and has shown positive evidence of seizure reduction by 26–40% in small RCTs (23–27) and up to 95% in smaller non-randomized trials (20). The largest RCT for DBS of the hippocampus (16 patients) showed that seven out of eight patients in the active therapy group had a reduction >50% in seizure frequency, and four of them became seizure-free (23). DBS to the centromedian nucleus of the thalamus (CMT) has also been studied, as the CMT projects widely to many cortical regions, especially frontal lobes (20, 28). Evidence from several small RCTs shows a better response to DBS of CMT in generalized epilepsy than focal epilepsy (27, 29).

Other DBS targets that have been explored for the treatment of epilepsy include the cerebellum (30), STN (31, 32), caudate nucleus (33), posterior hypothalamus (34), hippocampal fornix (35), and nucleus accumbens (36); however, the benefit of stimulation of these targets remains uncertain.

### DBS in Psychiatry

DBS offers a treatment option for resistant or severe psychiatric illness, often not responsive to oral pharmacologic agents and psychotherapy (37).

**Figure 3:**
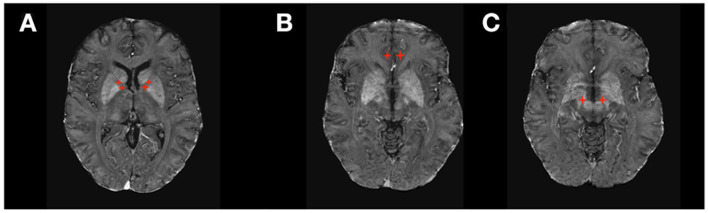
DBS targets in Psychiatry. **(A)** Ventral Capsule/Ventral Striatum (VC/VS) and Anterior Limb of the Internal Capsule (ALIC); **(B)** subcallosal cingulate gyrus including Brodmann area 25 (SGC25); **(C)** Subthalamic Nucleus (STN).

Prospective studies, retrospective reviews, and meta-analyses have demonstrated the efficacy of DBS in severe or extreme obsessive compulsive disorder (OCD) (38–40). Several targets, including ventral capsule/ventral striatum (VC/VS), anterior limb of the internal capsule (ALIC), and STN have been investigated for OCD (41, 42) (Figure 3). Outcomes are generally comparable across all implantation sites (Table 1), with an average improvement in OCD symptoms of 45% on the Yale Brown Obsessive Compulsive Inventory (53). Previously investigated predictors of outcomes, including presence or absence of hoarding disorder, intra-operative mirth or reflexive smile, and age at onset of symptoms, are inconsistent predictors of response and appear inadequate to inform clinical decision-making (38). Recent studies have investigated individualized DBS targeting for OCD using diffusion tensor imaging (DTI) and probabilistic tractography (54, 55). White matter pathways in the anterior limb of the internal capsule can be variable, and modeling the fibers may allow for localization of fibers of interest for OCD treatment, such as those connecting the nucleus accumbens and ventral striatum to target areas in the orbitofrontal cortex and medial prefrontal cortex (56).

**Table 1:** Summary of clinical data on deep brain stimulation for psychiatric indications.

**References**	**Study design**	***N***	**Target**	**Indication**	**DBS parameters**			**Follow up (months)**	**Outcomes (primary)**	**Outcomes (secondary)**	**Side effects**
					**Frequency (Hz)**	**Intensity (V/mA)**	**Pulse Width (mcS)**				
Abelson et al. (43)	Prospective blinded trial (on–off), open follow up	5	ALiC	OCD	130–150	5–10.5 V	60–210	4–23	1/5 with >35% reduction in YBOCS over baseline	Not defined	Mood changes, hypomania, transient sensory changes
Goodman et al. (44)	Randomized, staggered onset programming under blinded conditions	6	VC/VS	OCD	130–135	2.5–8.5 V	90–210	12	YBOCS response rate 66.7% after 12 months, no improvement during sham programming period	HAM-D, POMS improved in both groups, 4/6 improved on CGI	Emotional changes, hypomania
Denys et al. (39)	Open lead in, double blind cross over, on–off, open follow up	16	NAcc	OCD	130	3.5–5 V	90	21	YBOCS change- Open phase (mean 72% reduction), Double Blind (25% reduction mean active vs. sham)	Not defined	Mild forgetfulness, word finding difficulties
Dougherty et al. (45)	Multi-site sham controlled randomized trial with open label follow up	33	VC/VS	Depression	Not reported	Not reported	90 or 210	24	MADRS response rate not significantly different between active and sham (*p* = 0.53)	Not defined	Depression, insomnia, suicidal ideation, irritability, hypomania
Bergfeld et al. (46)	Open label optimization, randomized double blind, cross-over active/sham	16	ALiC	Depression	130 or 180	2.5–6 V	90	3 months open label, 4 weeks blinded cross-over, 1 year follow up	HAMD-17 difference active (13.6) vs. sham (23.1) (post open optimization phase) *p* ≤ 0.001	MADRS, IDS-C significantly lower active vs. sham (*p* = 0.001)	Mania, nocturia
Holtzheimer et al. (47)	Multi-site randomized sham-controlled trial	128	SGACC	Depression	130	4–8 mA	91	6 months randomized sham-control with 6 months open label follow up	MADRS response not significantly different active vs. sham (20, 17%)	Not defined	Head pain, infection, worsening depression, suicide attempt, death by suicide
Merkl et al. (48)	Double blind randomized controlled trial	8	SGACC	Depression	130	5–7 V	90	24	HAMD-24 baseline active vs. delayed onset 34.5, 32.5; at week 8 active vs. delayed onset 29.2, 31.2 (*p* = 0.291); at 24 months active vs. delayed onset 26.3, 15 (*p* = 0.49)	BDI-II most time points favored delayed onset with statistical significance, MADRS reductions at time points not significant	Hypomania, pulling sensation
Coenen et al. (49)	Double blind randomized controlled trial	16	SLMFB	Depression	130	3 mA (SD 0.5 mA)	60	12	MADRS reduction 29.6–12.9 at 12 months	HAMD-28 −2.23 *p* = 0.03	Strabismus
Barcia et al. (50)	Prospective randomized, double blind trial	7	NAcc and “striatal axis”	OCD	130	4.5	60	21	6/7 Y-BOCS reduction >35%, all stimulation (including sham) statistically significant reduction vs. baseline	Best contact varied between patients	No cognitive adverse effects of striatal stimulation, no seizures
Polosan et al. (51)	Randomized, double blind, within subject design	12	STN	OCD	130	“Individually adjusted”-parameters not reported	60	None	Ratings of Pleasantness/Arousal when viewing differently valenced images- OCD ON DBS more positive ratings, no interaction effect	Not defined	Not reported
Mosley et al. (52)	Prospective blinded trial (on–off), open follow up	9	BNST	OCD	130	4.5	90/120	15	Mean change YBOCS (blinded −4.9 points, *p* = 0.025), MADRS (blinded −3.4 points, *p* = 0.3)	7/9 patients >35% reduction in YBOCS after open label phase with CBT	1 IPG infection, 1 lead migration, psychiatric admission in setting of non-response, reduced libido

*OCD, Obsessive Compulsive Disorder; ALiC, Anterior Limb of Internal Capsule; VC/VS, Ventral Capsule/Ventral Striatum; NAcc, Nucleus Accumbens; SgACC, Subgenual Anterior Cingulate Cortex; SLMFB, Superolateral Medial Forebrain Bundle; STN, subthalamic nucleus; BNST, Bed Nucleus of the Stria Terminalis; HAM-D, Hamilton Depression Rating Scale; POMS, Profile of Mood State; CGI, Clinical Global Impression; YBOCS, Yale Brown Obsessive Compulsive Scale; MADRS, Montgomery-Asberg Depression Rating Scale; BDI, Beck Depression Inventory; IDS, Inventory of Depressive Symptoms; IPG, Implanted Pulse Generator; mA, milli-Amps; mcS, micro-seconds*.

Major depressive disorder is another psychiatric condition that has been studied using DBS (Table 1). Several industry-sponsored trials have yielded inconsistent results related to the efficacy of stimulation of VC/VS and anterior cingulate cortex (ACC) (45, 46). Smaller trials have reported remarkably rapid and efficacious effects of stimulation of the medial forebrain bundle (mFB) in depressive disorders (49, 57, 58). However, other trials have failed to find improvement (59). Selecting patients through biomarkers, including metabolism assessed through positron emission tomography, may improve response rates (60).

Case reports and case series have examined the use of DBS in other psychiatric conditions with severe and persistent symptoms, including anorexia nervosa, schizophrenia, bipolar disorder, and opioid use disorder (61–63). Results for these populations, who often lack evidence-based treatments for resistant illness, have been promising. Individualized targeting, including tractography and monitoring for effect using sensing technologies, may help clarify candidates and maximize therapeutic outcomes.

Of relevance, a local field potential (LFP) study in post-implantation patients revealed increased alpha power in major depressive disorder but not OCD patients (64). No significant variance in the beta band power was reported in either condition. A case reporting a patient with sensing DBS and cortical epidural stimulation reported elevated theta power in VC/VS (65). STN stimulation of OCD patients revealed increased coupling with other relevant locations [STN-anterior cingulate cortex (ACC)] in different power bands, including beta, alpha, and theta (66). Therefore, the specific role of each oscillatory frequency and measure (power, coherence) remains to be elucidated, but LFP monitoring appears to be a promising assessment tool for DBS psychiatric indications.

## Innovations in DBS Technology

### Hardware Innovations

Multiple features have changed in the DBS hardware over the last few years. The industry is evolving toward flexible extensions that provide pliability and stretch in connecting the lead to the impulse generators (IPG), which themselves now have contoured edges to reduce skin pressure and pliable headers. Newer IPGs are current driven so that the amount of energy delivered does not depend on the variability in patients' impedances. The pulse width can be reduced to a minimum of 20 μs to selectively stimulate small-diameter axons to reduce the incidence of dysarthria and capsular side effects (67–69). In addition, certain systems have the capability of multiple independent current control (MICC), which allows each contact to have a separate current source. This offers the possibility of fractionalizing current over multiple electrodes at the same time or even using two different frequencies on the same DBS lead.

The introduction of “directional leads” characterized by the middle two (of four) rings divided into three segments has allowed generating an axially asymmetric volume of tissue activation (VTA) tailored to the individual patient's anatomy (67, 70). DBS bioelectrical parameters, such as therapeutic impedance and surface current density, are highly dependent on the electrode surface and inevitably change when switching from a circular ring contact to a single-segment activation. Therefore, the change from “conventional” to “directional” stimulation requires a few adjustments. The intensity needs to be lowered to prevent an excessive increase in current density (current intensity/electrode surface). For this reason, stimulation adjustments should be performed using a smaller step-size amplitude (0.1–0.3 mA as compared to the traditional 0.5 mA). In addition, since the electrical current tends to flow out of the electrode through its edges, the lateral VTA extension is wider than the surface covered by the electrode, and this needs to be taken into consideration as the simultaneous activation of multiple adjacent segmented electrodes can lower the extent of directionality (71). A recent retrospective review of DBS cases showed that the vast majority of STN-DBS PD patients (74%) and Vim-DBS ET patients (79%) implanted with directional leads were actively using directional over conventional stimulation settings (72). In addition, the recent approval of a 4-port, 32-contact DBS device (Vercise™ Genus, Boston Scientific, USA) has introduced opportunities for DBS leads with a greater number of directional electrodes. A clinical study (eXTend 3D, ClinicalTrials.gov Identifier: NCT04577651) is currently testing this novel 16-contact directional leads (Cartesia X/HX; Boston Scientific) to determine whether the additional directional rows on these leads can further improve directional programming.

Finally, novel DBS waveforms using time-varying high-frequency pulse trains are under investigation. This technology aims to desynchronize pathological neural circuitry more effectively than conventional programming using a lower amount of energy, potentially resulting in cumulative and long-lasting therapeutic benefits (73, 74). If preliminary data are confirmed, this technology may translate into less frequent battery replacements and superior clinical benefit. Along the same line, a novel DBS system has been developed (Pinstm DBS, Beijing Pins Medical Co., China) to deliver variable frequency stimulation (VFS) using two distinct frequencies (i.e., 80 and 130 Hz). Preliminary studies evaluating VFS in PD patients treated with STN DBS have shown improvement in freezing of gait compared to traditional stimulation settings (130 Hz) (75–77). A clinical study of intermittent, desynchronizing, coordinated reset pulse trains is also underway using investigative devices based on commercially available DBS systems (Reset-DBS, Clinicaltrials.gov identifier: nct03732898).

### Software Innovations

To assist with the challenges associated with more complex programming strategies, DBS producers have developed supportive software platforms. Abbott has taken a strictly functional programming approach and developed simple monopolar review documentation and decision-support software tool (Informity™) that aims to simplify directional programming based on response to stimulation (70). The system guides clinicians to determine the therapeutic window (TW) for each electrode and automatically calculates the appropriate step size for incremental stimulation intensity changes for directional stimulation. Then, an integrated decision tool allows sorting the montages based on factors such as power, TW, or TW percentage (TW%), which is computed as the ratio of TW to therapeutic current strength (TCS). A final sorting option, balanced threshold, provides an additional level of optimization to balance gains in TW% while minimizing power consumption. Boston Scientific has supported a revised version of the monopolar survey called “non-zero amplitude programming,” based on the approach first described by Steigerwald et al. (67). Instead of sequentially testing each electrode for efficacy and adverse effect thresholds, the examiner can gradually move a predefined electrical field along the vertical axis to determine the level of maximal effectiveness. Once the best vertical level of stimulation has been identified, the VTA can be moved on the horizontal axis to determine the best direction of stimulation. This method and others present opportunities for automation and algorithms to simplify the workflow.

**Figure 4:**
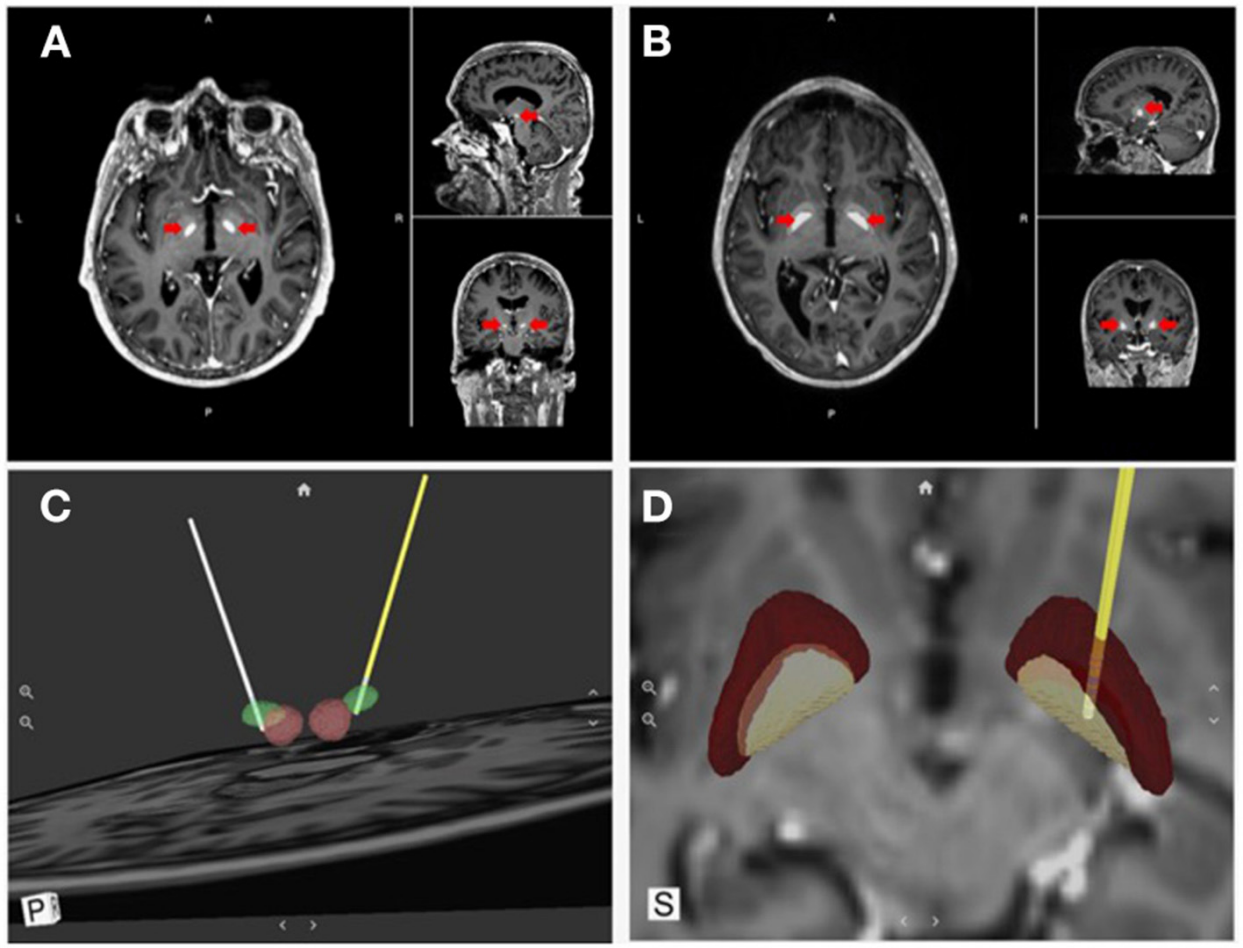
Direct visualization of DBS targets and lead location. Surgical Information Sciences (SIS), provides a patient-specific model of DBS targets using the patient's own enhanced clinical MR image. **(A,B)** shows alpha maps visualizing the STN and GPi, respectively (red arrows); **(C,D)** show 3D reconstructions of the intended targets registered with the DBS lead location in respect to the anatomical structures (STN & GPi, respectively).

Visualization tools have also been developed to support an “image-guided” programming approach based on reconstructing the patient-specific anatomy from magnetic resonance imaging (MRI) and computerized tomography (CT) data. One such visualization tool, developed by Surgical Information Sciences (SIS), provides a patient-specific, 3D anatomical model of specific brain structures using the patient's own enhanced clinical MR image (Figure 4). The technology makes use of the fact that some structures in the brain, specifically the STN and the lamina/border between the GPI and internal capsule (IC), are not easily visualized in 1.5T or 3T standard clinical MRI but are better visualized using high-resolution and high-contrast 7T (7 Tesla) MRI (78). The SIS approach uses pre-trained deep learning neural network models, based on these ultra-high-resolution 7T MRIs, to be applied to a patient's clinical image to predict the shape and position of the patient's specific brain structures of interest (i.e., STN or GPi/GPe) (79, 80). The output of the SIS System can then be used for planning stereotactic surgical procedures. The company has validated the accuracy of the STN and GPi predicted locations within 1 mm of the ground truth locations (81). The system can locate and identify implanted leads visible in post-operative CT images and present them in a 3D model in relation to the predicted brain structure from the preoperative processing. These unique features can provide feedback to the surgeon regarding the final location of the lead compared to the surgical plan, as well as inform the programming neurologist about the lead location and contact orientation in relation to anatomical and physiological targets to reduce programming complexity. Visualization tools also allow modeling the VTA generated by various stimulation settings to identify the ones with the highest probability of success (maximal benefits and minimal side effects). Examples include Medtronic SureTune™ and Boston Scientific GUIDE™ XT, which use a patient-specific atlas to provide visualization. Potential shortcomings are represented by the inability to account for individual electrode impedances, axons orientation, and brain tissue inhomogeneity. These tools' accuracy is also limited by axial rotation or vertical migration of the DBS lead that may occur after the image has been acquired.

Finally, a recent software innovation available on the Abbott system has allowed patients to download an app to turn their iPhone into a controller device so that their mobile phone can be used to manage their DBS therapy without the need for a separate patient controller device. Those patients who don't have an iPhone receive an iPod Touch as their controller device.

### Telemedicine in Movement Disorders

Telemedicine has been tested in several forms over the last decade in the evaluation and care of movement disorders patients (82, 83). More recently, as a result of the pandemic, telemedicine gained broad acceptance in the movement disorders community. Interviewed patients reported reduced travel, wait time, stress and expense, and greater comfort, convenience, and access to specialists (84).

**Figure 5:**
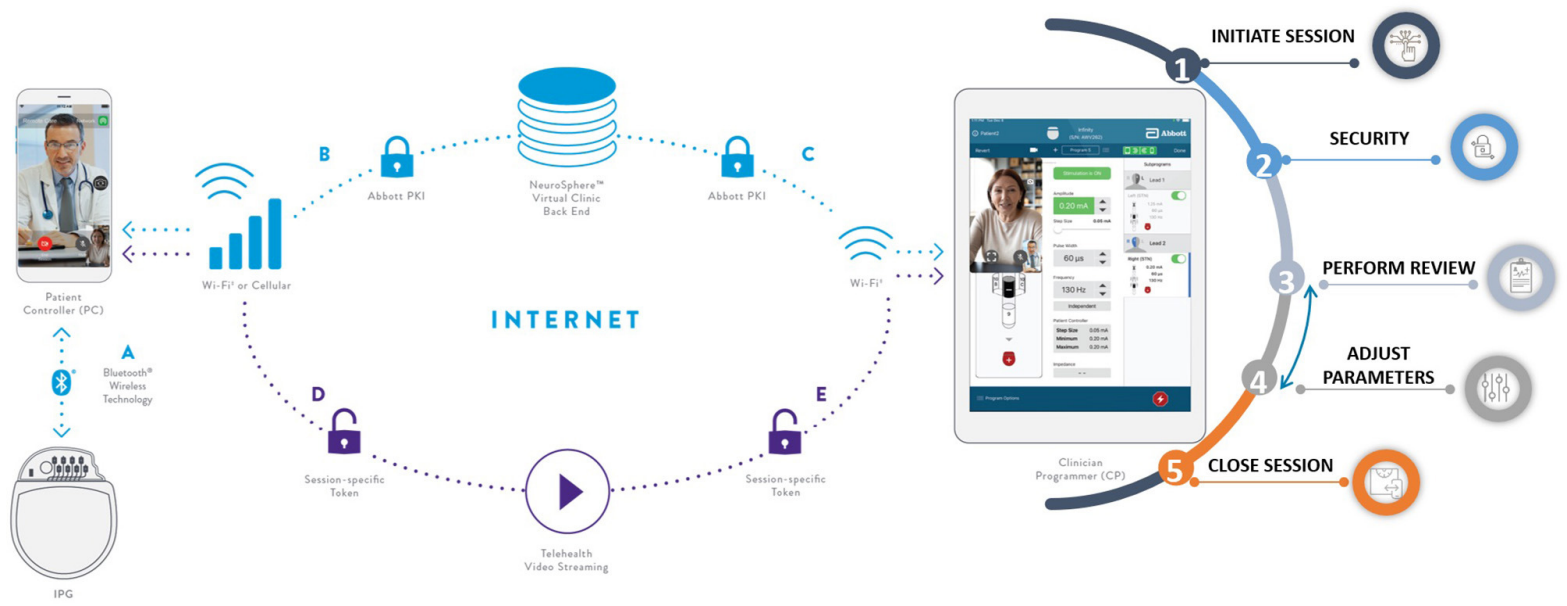
Abbott's NeuroSphere™ Virtual Clinic. The software platform enables the remote DBS clinician to not only investigate the patient's IPG and run system checks remotely but also to make stimulation therapy changes over the internet.

Abbott recently received FDA approval for a complete DBS telemedicine solution to enable both video conferencing telemedicine and the remote clinician's ability to connect to the patient's IPG over the internet to make stimulation (therapy) changes (NeuroSphere™ Virtual Clinic; Figure 5). This solution was delivered as a software update without the need for change in implanted hardware (leads or IPG) to ensure that access to remote programing is available to every patient implanted irrespective of when they were implanted. Once the remote programming session starts, the platform first copies the stimulation settings of the active program delivering therapy to the patient and locks it into what is referred to as the protected recovery program. This failsafe mechanism ensures that if the remote programming session disconnects as a result of internet disruptions or due to incorrect termination of the session, the patient's IPG automatically goes back to the original therapeutic settings from the start of the remote session. This is referred to as the “protected recovery program.” During the remote session, clinicians can check system and program impedances, check the battery status, change stimulation parameters, manage (add/delete/edit) programs, and even generate a session report for telemedicine reimbursement documentation.

### The Upcoming Era of DBS Sensing Technology

**Figure 6:**
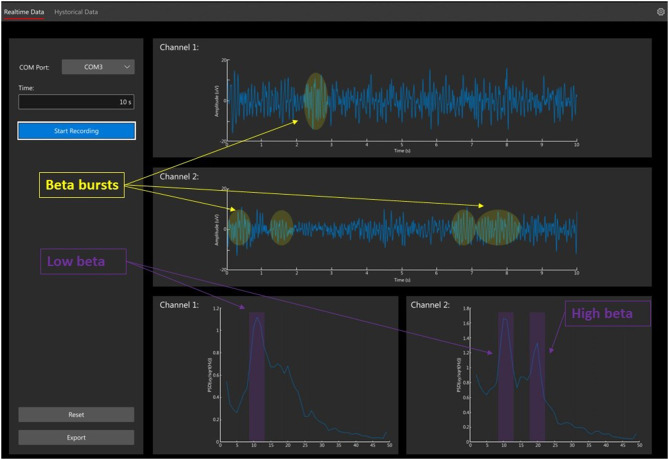
Local field potential recordings (LFPs). The figure shows the recording from *n* = 2 subthalamic nuclei in a patient with Parkinson disease. The raw LFP signal of a 10-s recording is presented in the 2 top traces. The frequency spectra are displayed in the 2 bottom graphs. LFPs are recorded and filtered with 8th poles low pass filter, corner frequency at 40 Hz, for stimulus artifact suppression (AlphaDBSvext, Newronika, Italy). The device streams the signal *via* radiofrequency to the receiver connected to the computer. Beta bursts activities are highlighted in yellow with low and high beta peeks in purple.

Sensed brain signals represent a new opportunity for advancing the standard of care in DBS therapies, as briefly mentioned in the DBS in Psychiatry section. Historically, access to such signals was limited to the use of investigational devices, which allowed constrained exploration of signals in research contexts (85, 86). In recent years, this work has extended to chronically recorded LFPs measured from the macro electrodes used for stimulation. Rather than the single-unit firing information from the microelectrode, these potentials represent the compound activity of several individual units which can be influenced by multiple variables, including neuronal and synaptic sources, recording volume, surface of the recording electrode, and electrode-tissue interface impedance (87, 88). In a widely accepted computational technique, LFP is simulated as the sum of all excitatory postsynaptic currents plus the inhibitory postsynaptic currents in the recording region (89). The amplitude of these signals is in the order of units of microvolts (90) when recorded from subcortical structures, but it can be significantly higher (tens of microvolts) when recorded from the cortex using electrode strips (91). In contrast with the frequency band of single-cell activity, which ranges from 500 Hz to 5 kHz, the LFPs frequency band is 2–100 Hz, quite similar to common electroencephalography (Figure 6).

Considering the feasibility of recording LFPs (92), three clinical scenarios can be imagined for this neurophysiological biomarker: (a) if signals correlate with the patient state, they may be useful to provide objective measures of outcomes outside of the clinic; (b) if signals respond to therapeutic interventions, they may be helpful to optimize therapy and guide programming in the clinic; and (c) if signals both correlate with patient state and respond predictably to therapy, then they may finally be useful in implementing closed-loop systems that adapt to changes in symptoms over time. It must be noted, however, that the consistency of oscillatory biomarkers among subjects remains to be confirmed (93).

Exploring the first scenario, multiple groups have shown that specific oscillatory rhythms, such as enhanced synchrony in the beta band (13–30 Hz), often correlate well with patients' symptoms (especially akinetic and rigid symptoms of PD) (94). This may allow such measures to be used as a correlate of the patient state outside of the clinic, helping quantify the occurrence and severity of symptoms in a real-world context. It might be expected that a signal such as beta that correlates with akinetic/rigid PD features might show fluctuations associated with a patient's On/Off cycling (95). This might allow a clinician to understand medication dynamics (wash-in, wash-out), differences in cycles across circadian rhythms (wind up or accumulated medication effects at the end of the day), compliance issues with medication dosing or timing, and perhaps the exploration of more complex drug/stimulation interactions. Further, capturing a neural signature during a patient marked symptomatic event outside of the clinic may help a clinician interpret that patient report, as the signature for a break-through tremor event may be expected to differ from other potentially confounding events such as atypical dyskinesias (95).

Toward the second scenario, such signals have also been shown to respond to, and correlate with, the onset and effectiveness of DBS and oral medications (96). This may allow such signals to be used to optimize stimulation settings in clinic, for example, by providing a real-time view of response during a titration process. Such signals, recorded chronically outside of clinic, might also prove useful to configure therapy over time by providing objective measures to compare different therapy regimes, identify or troubleshoot break-through symptoms, or assess variability in response across different circadian or medication states. As more specific examples, a signal that correlates with symptoms (e.g., beta band) may be helpful to review as stimulation amplitude is titrated on a specific electrode in a specific hemisphere (97). This may help identify a threshold amplitude, the first at which suppression of the signal of interest is observed. It may also help identify an amplitude beyond which further increases no longer yield additional suppression, thereby suggesting a practical upper limit of stimulation on this contact. In addition, if measures of stimulation side effects can be identified, observing these measures in clinic as parameters are changed may help avoid a scenario in which a patient leaves the clinic in an overstimulated state. As a specific example, gamma-band oscillations have been shown to correlate with dyskinetic states (98); observing neural signals during stimulation titration for the emergence of gamma peaks may prove useful in avoiding dyskinetic side effects outside of the clinic. Although evidence is more limited, it may also be the case that the location of signals of interest like beta relative to lead electrodes may help inform the best clinical contact to use for programming, thereby reducing the complexity of configuring a patient by providing guidance on contacts more likely to be efficacious (99).

Finally, if LFPs are indeed robust and broadly available, correlate well to cardinal symptoms of diseases like PD, and ultimately respond predictably to stimulation and medication, it may be feasible to apply closed-loop methodologies using these correlations to adapt therapy over time and thereby adjust for fluctuations in symptoms (100). In multiple studies, such closed-loop methodologies have been shown to be energy-saving, to reduce the prevalence or likelihood of stimulation-related side effects, and finally to promise better overall motor symptom efficacy (101). A limitation of these studies is that they have all occurred in clinic and for relatively short periods Larger scale studies outside of the clinic setting will therefore be necessary to confirm the safety, performance and potential benefits of closed-loop systems. In fact, from a technology viewpoint, implementing LFP-based, closed-loop adaptive control is challenging because the simultaneous recording of LFPs during DBS delivery is inevitably affected by a stimulation artifact in the range of 100–200 mV. Therefore, conventional amplifiers cannot interpret LFPs during simultaneous stimulation. Specific strategies, such as high-order analogic filtering (102), active charge-balanced stimulation, input blanking, peristimulus sampling, and software data manipulation (103, 104) are required to allow simultaneous recording/stimulation. To this purpose, specific amplifiers for external recording have been introduced at a research level (105) and in platforms intended for commercial use (AlphaDBSvext, Newronika, Italy).

**Figure 7:**
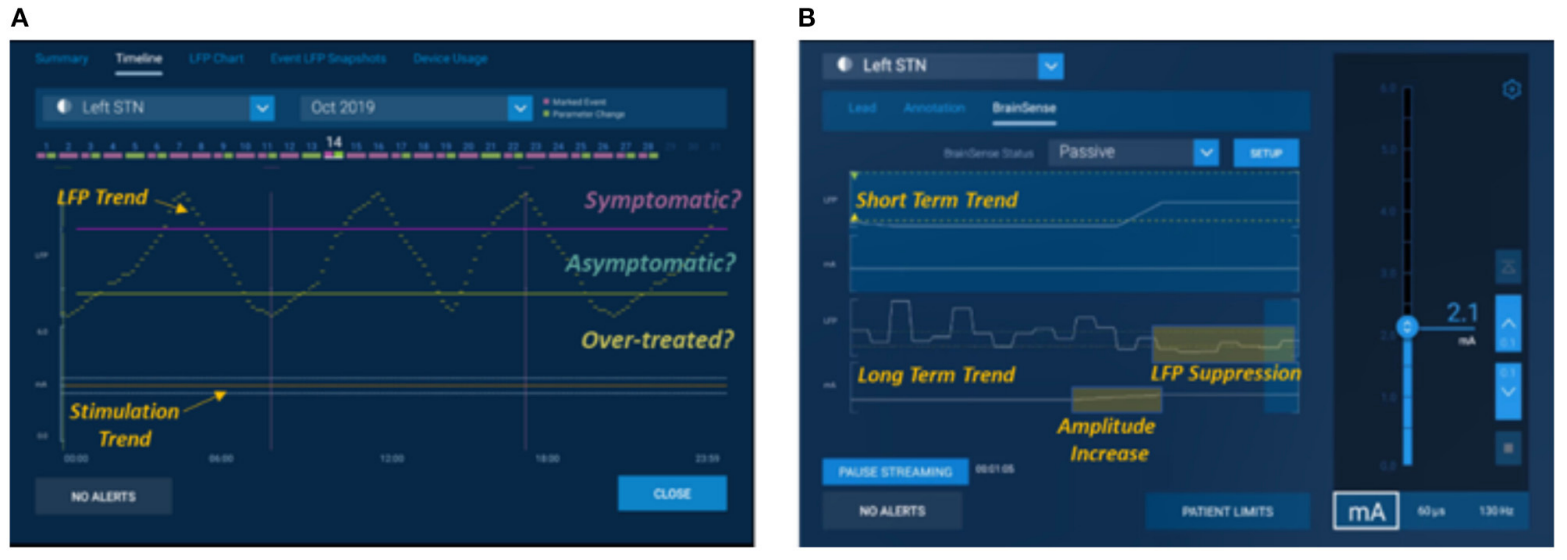
Medtronic's Percept™. Examples of use cases of local field potentials from Percept PC™ programming interfaces using exemplary representative data. **(A)** shows a signal of interest such as average beta power trended over long periods to show fluctuations and times when a correlated symptom might present: “Symptomatic,” periods where a symptom might be absent: “Asymptomatic,” and periods where a patient may be over-treated and at risk of side effects: “Over-Treated,” and **(B)** shows use of signal viewed in real time to understand how it responds to amplitude titration during in clinic programming, in this example evoking a suppressive effect subsequent to a stimulation increase.

With the first availability of commercial devices (Percept PC™, Medtronic, USA) in the movement disorders field to chronically sense brain signals, these opportunities have become more broadly available (Figure 7). Specifically, this commercial system supports features that enable aspects of the first two scenarios described above—(1) chronic objective measures available inside and outside of clinic, although this is limited to a relatively short (~1 h) of LFP recordings per week to preserve battery longevity, and (2) real-time signals in clinic to guide parameter configuration although this is restricted to bipolar recording, which may limit the spatial resolution of the LFPs. Very early evidence (106, 107) suggests that the research community's findings can be replicated in these commercial devices, with early publications showing the feasibility of using in-clinic signals in the programming process and at-home signals to understand the real-world experience outside of the clinic. The final use case, closed-loop or adaptive therapies outside the clinic, requires more exploration and evidence. In parallel to the Medtronic system, also the AlphaDBS system has closed-loop adaptive capabilities, currently under clinical investigation (ClinicalTrials.gov NCT04681534). In addition, Percept PC™ is enabled for additional control algorithms through a software unlock. These capabilities will be assessed *via* industry-sponsored studies in PD (e.g., ADAPT PD) underway as of early 2021.

While LFP sensing represents one of the most exciting innovations in the field, the limitation of currently available sensing technology to overcome technical challenges related to artifacts caused by the cardiac rhythm, stimulation, and movements remains a challenge. These factors can interfere with the power spectral density at the corresponding frequency and its ascending harmonics. The use of wearable sensors represents alternative options to LFP-based closed-loop DBS. A recent work implementing closed-loop algorithms supported by wearable sensors demonstrated that automated programming software could achieve equivalent efficacy to conventional programming in fewer steps (108).

## Innovations in the Surgical Approach

### Robotic-Assisted Surgery

**Figure 8:**
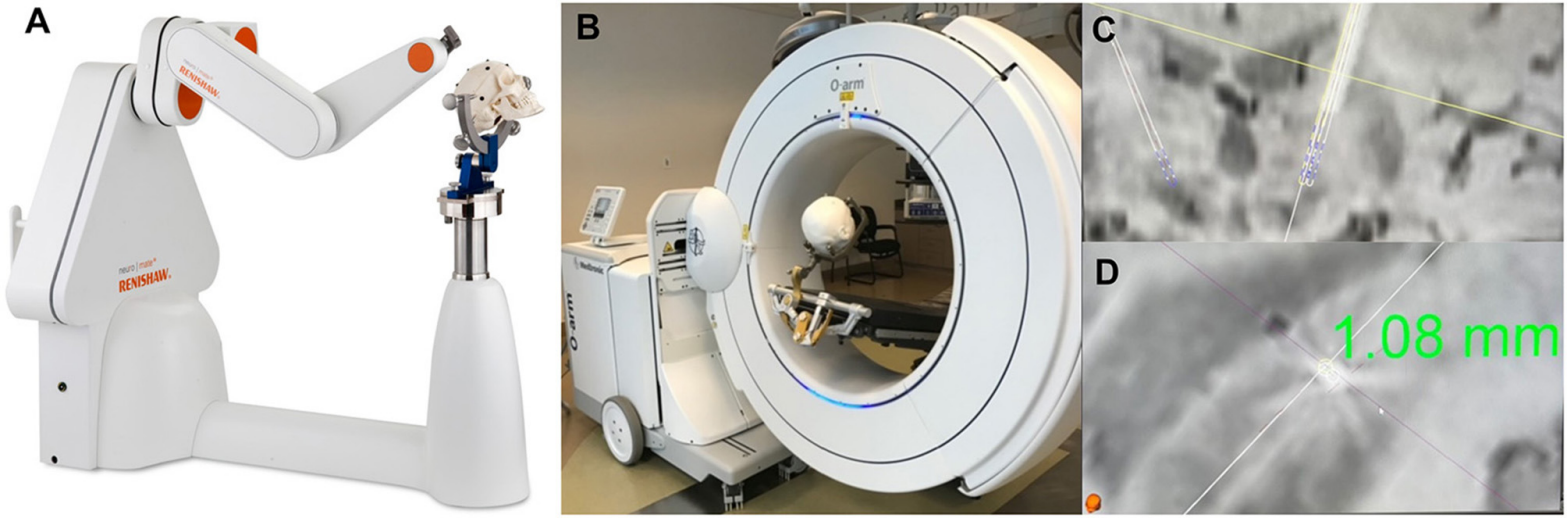
Robotic Surgery assisted by Intraoperative Imaging. The utility of combining intraoperative robotic technology with intraoperative imaging allows for real time evaluation of accuracy of lead placement regarding the preoperative planned trajectory. This allows for adjustment of the cannula trajectory prior to MER recordings or lead placement. **(A)** Renishaw Neuromate robot. **(B)** Medtronic O-arm with mock set-up for acquiring intraoperative imaging. **(C,D)** Intraoperative O-arm image fused with preoperative SWI MRI showing a 1.08 mm lateral deviation from the planned trajectory.

A new generation of robotic tools is rapidly emerging to further improve the surgical accuracy of DBS, brain biopsies, and therapeutic ablations (Figure 8). The first experience with robotic use in neurosurgery dates to 1985, when the PUMA (Programmable Universal Machine for Assembly) 200, a device initially developed for industrial use, was used for a stereotactic brain biopsy (109). This was followed by the NeuroMate system (Integrated Surgical Systems) in 1987, then by the ROSA Brain system (Medtech, Zimmer Biomet) and the Renaissance robotic system (Mazor Surgical Technologies), both of which received FDA approval for intracranial use in 2012 (109).

The primary goal in DBS surgery, where millimeter accuracy is crucial, is to minimize the error in the placement of DBS leads. Several factors may contribute to the inaccuracies of stereotactic operations, including the quality of pre-operative and intra-operative registration imaging, image merge error, accuracy of frame registration, frame vs. frameless utilization, microelectrode drive accuracy, deformation of the stereotactic frame due to overtightening of the fixation pins, longstanding use or changes from repeated sterilization processes, errors in manual input of X, Y, Z coordinates, intra-operative imaging quality, and post-durotomy time among others (110). Although many of these variables are non-modifiable, one of the largest areas where error can be minimized is manually setting the frame coordinates. This can be improved by utilizing a robotics-based stereotactic positioning system to improve both the accuracy and the precision of lead targeting. While robotic systems have their own inaccuracies, calibration algorithms built into the devices reduce inaccuracies and remove inherent human error (111). All robotic devices also have methods to verify targeting precision after image acquisition, merge, and registration sequences, allowing for modification of the target plan based on inaccuracies in targeting precision.

A recent meta-analysis compared various DBS implantation techniques from 27 studies over 16 years and found that the pooled mean targeting error was 1.91 mm across all modalities. They also found that robot use was associated with a mean reduction in targeting error of 0.79 mm (112). Various studies have reported outcomes utilizing the different robotic technologies. Neudorfer et al. compared the ROSA robotic system to conventional frame-based targeting and found a mean error of 0.76 ± 0.04 mm with the ROSA robot compared to 1.11 ± 0.07 mm with frame-based targeting. They also reported that with the ROSA robotic system, no leads errors exceeded 1.52 mm. In comparison, 21.3% of frame-based placement exceeded this value, and 8.75% of leads placed with conventional frame-based targeting had errors of >2 mm (113). This is notable as with STN placement, lead deviation over 1 mm has been shown to be more likely associated with unwanted stimulation-induced side effects (114, 115). Similar loss of effectives with target deviation were shown for VIM DBS as well (116). Neudorfer et al. also reported a significant reduction in operative time between frame-based and robotic targeting of 394.8 ± 66.6 min and 280.5 ± 59.2 min, respectively (113). This decreases post-durotomy time, CSF loss and brain shift, and anatomical distortion (117–119). Ho et al. have utilized the Mazor Renaissance robotic device and report a mean radial error of 1.4 ± 0.11 mm (120). Moran et al. report on 226 trajectories using the NeuroMate robotic system with asleep DBS technique with a mean radial error of 0.6 ± 0.33 mm and Euclidean error of 0.78 ± 0.37 mm (121).

Overall, a growing volume of literature demonstrates the non-inferiority of robotic DBS targeting vs. frame-based DBS targeting and frequently suggests more accurate and precise targeting with robotic solutions. Therefore, many centers are adapting robotic targeting and other modalities to advocate for the improved safety of asleep-based DBS lead placement.

### Asleep DBS

The interest in asleep DBS has grown over the last few years due to the potential advantages of improved patient comfort. The definition of asleep DBS remains controversial as it includes several different procedures grouped by the common denominator of being performed under general anesthesia. Asleep DBS can be performed with a variety of techniques; for example, asleep DBS can mean the patient is under heavy sedation with no intraoperative neurophysiologic recording possible, vs. light sedation allowing for interoperative recording. Along these lines, researchers have used these various scenarios during clinical studies, with some comparing outcomes of “asleep DBS + physiology” with those of “awake DBS + physiology” (122, 123), whereas others compared “asleep DBS—physiology” with “awake DBS + physiology” (124–126). It is important to note these differences exist when interpreting results of large systematic literature reviews (127, 128) in which cohorts are grouped according to the level of consciousness, awake vs. asleep, and not according to the surgical technique. For example, one meta-analysis found similar efficacy for asleep and awake DBS. Still, half of the included asleep cohorts received microelectrode recording (MER) (upwards of 5 tracks), and some even received macro-stimulation to check for capsular side effects. Therefore, particular attention is warranted to avoid running the risk of incorrectly extrapolating these results to all asleep DBS methods as some techniques for asleep procedures are more thorough than others.

One of the main driving forces allowing for asleep DBS is the ability for high-quality intraoperative imaging. The two main sources for this are intraoperative MRI (iMRI) and intraoperative CT (iCT). While both have proven to be accurate, there are important differences to note between them. The ClearPoint system (129) uses an iMRI aiming device with sub-millimetric application accuracy to give the surgeon real-time feedback when planning the electrode placement trajectory. Targeting can also be adjusted after the dura is opened, in other words, after brain shift has occurred due to cerebrospinal fluid loss and pneumocephalus. The shift of basal ganglia structures during burr-hole-based procedures averages 0.6 mm, but is >2 mm in 9% of patients (130). In addition, iMRI obviates the need for image fusing and thus avoids potential merging errors. In comparison, iCT guided surgeries have more factors involved that may lead to a higher risk of targeting error. iCT is often performed with a traditional stereotactic frame which is associated with an application accuracy exceeding 1 mm (1–3 mm) (131). In addition, it requires merging of CT with pre-operative MRI for targeting, which is associated with an average fusion error slightly more than 1 mm (132, 133). The difference occurs when a second merge takes place intraoperatively to confirm lead placement. By this time, pneumocephalus and CSF loss may have deformed cerebral structures which can contribute to lead placement error with this technique. There can also be distortion of the iCT images secondary to the quality of the iCT modality or metal artifact from the frame or surgical instruments that can affect image fusion or accurate interpretation of lead placement. Experienced centers use strategies to minimize these errors and have indeed reported good outcomes with iCT (125, 134). Nonetheless, differences in imaging methodology should be kept in mind when discussing outcomes of asleep DBS.

In terms of accuracy, some studies showed that the accuracy obtained with asleep DBS is higher than with awake surgery (135), whereas others found no difference (124, 127, 136, 137). A recent study found no difference in radial error between microelectrode recording (MER)-guided electrode implantation in awake surgery, and iCT-guided electrode implantation in asleep surgery (124, 137). Of note, it is not always clearly described how the error is measured in awake cases, when the surgeon intentionally places the DBS lead away from the intended target because of MER or test-stimulation findings. This will increase the distance from the initial target, and therefore the error may be larger depending on if this correction was not accounted for when calculating the error. In asleep cases, without physiology there would be no rationale to move the lead, and therefore the error would be smaller.

In addition to the technical variability in asleep procedures, most studies suffer from lack of randomization, lack of blinded ratings, retrospective design, lack of controls, use of historical/inappropriate controls, statistical under-powering, short follow-up, high attrition rates, and more. The ongoing GALAXY study is an RCT of asleep vs. awake DBS, with both arms receiving MER (138). Another promising example is a randomized, non-comparative pilot study of robot-assisted, iCT guided DBS of the STN under either general anesthesia or local anesthesia with MER. This phase 2 pilot study is anticipated to lead to a larger randomized trial, the PARKEO 2 trial in France (137).

In conclusion, definitive data regarding the differential efficacy and consistency of various asleep DBS techniques and how each compares to awake, physiology-supported DBS, or to a hybrid of the two are still lacking, although the field is in rapid expansion.

## Innovations in Imaging for DBS

Advanced imaging techniques aim to improve the visualization of DBS therapeutic targets. Specific MR sequences such as quantitative susceptibility mapping (QSM) are being tested to improve DBS direct target visualization. In parallel, neuroimaging sequences such as diffusion MR imaging offer an unprecedented visualization of brain connections relevant to DBS safety and efficacy. Together, these neuroimaging advances aim to improve stereotactic targeting for awake and asleep DBS, potentially optimize DBS programming, and eventually assist in patient selection (139). Conventional DBS planning defines the stereotactic target location relative to specific brain landmarks, such as the commissures and the ventricles (140). While the advent of high-field MR and ultra-high-field (7T) has dramatically improved the visualization of internal brain landmarks (141, 142), the high iron content of the STN can cause significant MRI imaging distortion in T2 fast SE (FSE) sequences with conventional magnets, leading to an error of up to 2.4 mm, particularly evident at the nucleus periphery, where the borders remain obscure (143, 144).

The use of diffusion MR, on the other hand, allows visualizing white matter tracks relevant for the DBS targeting, opening the way to improved therapeutic targeting within the brain networks of interest. This technique not only identifies fibers relevant to DBS targeting but also differentiates them from those associated with stimulation-induced side effects, such as the internal capsule and the medial lemniscus.

### Neuroimaging of the GPi

The GPi is a challenging target due to its complex functional anatomy. For example, GPi stimulation effects are site-specific such that the stimulation of the ventral GP improves levodopa-induced dyskinesia, while dorsal stimulation can induce dyskinesia (145). Therefore, it is critical to distinguish clinically relevant GPi subregions to inform stereotactic targeting during DBS surgery.

Dedicated imaging such as the fast gray matter acquisition T1 inversion recovery sequence (FGATIR) has allowed delineation of the GPi nucleus (146). In efforts to further define the GPi subregions, neuroimaging-based parcellation of the GPi was performed in healthy control and movement disorder patients using 7T MRI (147). However, accurate parcellation is not always possible in conventional clinical magnet due to challenges such as limited image resolution and signal-to-noise ratio combined with the high density of short-range efferent and afferent connections between the GPi and the thalamus and striatum, the proximity to the fibers of the cerebral peduncle, and the loss of diffusion signal due to white matter degeneration in neurodegenerative disorders. These limitations notwithstanding, the GPI connections have been explored using high-field magnets, showing that the caudal-lateral GPi is connected with the putamen, while the posteroventral GPi is connected with the STN and ventral thalamus (148, 149), representing the ideal target for GPi-DBS. These findings are confirmed by the observation that the highest LFPs beta power (5–35 Hz) is recorded from the postero-lateral “sensorimotor” GPi region (150). Additional efforts are underway to better define motor from non-motor GPi sub-regions to aid stereotactic targeting during asleep and awake DBS.

### Neuroimaging of the STN and Its Surrounding Tracts

The STN is a critical hub in the basal ganglia with a tripartite functional organization. It has a sensorimotor area located posterior and dorsally, an associative area in its central part, and a limbic area in the most anterior and ventral region (9). With high-resolution imaging at 7T MRI combined with advanced post-processing techniques, it is now feasible to visualize these functional domains in individual patients (151, 152). These new capabilities help explain the importance of lead location relative to these functional STN domains, which in turn can provide crucial information for efficient DBS programming (153). The STN is close to multiple critical white matter tracts, including the corticospinal tract laterally, the medial lemniscus posteriorly, and the oculomotor tract ventromedially. STN has abundant connections to the motor cortex (primary motor, premotor, supplementary motor), non-motor cortex, and basal ganglia. These dense white matter bundles have crossing fibers specifically at the STN's periphery, making it challenging to use diffusion MRI for direct STN targeting. However, using optimized MRI sequences (30 diffusion gradients or higher and voxel size 2 mm), the white matter tracts in the subthalamic region (STN and its surrounding fibers system) can be visualized (154).

The proximity of DBS electrodes to certain white matter tracts in the subthalamic region makes specific locations more attractive for stimulation than others. Akram et al. (155) showed that DBS stimulation volumes with greater connections to the prefrontal cortex and supplemental motor area were more beneficial for rigidity, while those with connections to the supplemental motor area only were associated with improved bradykinesia and connections to the primary motor cortex with tremor improvement. It is also recognized that white matter pathways outside the STN boundaries may be relevant to the STN DBS beneficial effects. Given the tract-specific clinical effects, automated algorithms have been developed to identify STN-DBS locations most likely to yield good clinical outcomes (156). More research is required to test whether these neuroimaging advances can improve stereotactic targeting and the selection of optimal stimulation parameters.

### Connectivity-Based Imaging of the Thalamic Targets

Since the VIM boundaries are not readily seen on standard-of-care structural imaging, several groups have sought to use diffusion MRI to visualize the VIM. In tracing studies, the VIM is a functional relay between the cerebellum and the primary motor cortex, connected *via* the DRTT (157). Klein et al. showed that DBS locations that significantly reduced tremor were more likely to be in the proximity of the DRTT (158). Using patient-specific preoperative diffusion MRI and postoperative CT, other authors tested the relationship between DBS electrode proximity to the DRTT and clinical efficacy (159, 160). Since the DRTT is readily seen with both deterministic (161) and probabilistic methods (162), diffusion MRI was also incorporated in stereotactic targeting for direct and indirect VIM visualization (163, 164), and a tractography-based stereotactic targeting approach to the VIM has been recently described (161). Using this technique, the authors demonstrated high concordance between the location and extension of the VIM identified *via* tractography and MER (165), with potential improvement in surgical outcomes and integration in asleep procedures (156).

## Concluding Remarks

While new hardware and software capabilities in DBS technology promise to improve the accuracy of stimulation paradigms and expand the field to new neurological and psychiatric indications, innovations in imaging and surgical tools are helping remove some of the historical barriers to DBS adoption. Simultaneously, the explosion of technological advances in telemedicine opens up several new frontiers to advance remote DBS programming, reducing the burden of traveling to tertiary referral centers. Altogether, these innovations are anticipated to significantly impact the field of neuromodulation and lay the foundation for a new era of brain-technology interface. The extent to which these different implantable devices, surgical techniques, and asleep vs. awake procedures might impact short- and long-term DBS outcomes, however, remains to be clarified.

## Author Contributions

AM and BD: conception, organization, and execution of research project, writing of the first draft, and review and critique of manuscript. JS, KR, BC, SG, LR, SP, SC, NH, VK, AS, FS, and LV: writing of sections of the first draft, review, and critical intellectual contribution to the preparation of the manuscript. All authors listed above gave their final approval of this manuscript version.

## Conflict of Interest

AM has received support from the NIH (KL2 TR001426), speaker honoraria from CSL Behring, Abbvie, Abbott, Theravance, and Cynapsus Therapeutics. He has received a salary as chief Editor of Frontiers in Neurology, Experimental Therapeutics, and grant support from Lundbeck and Abbvie. BC has received speaker honoraria from Abbvie. SG is an employee at Medtronic. LR is an employee and shareholder in Newronika, Inc. SP is an employee of Abbott Laboratories. SC is an employee of Boston Scientific. NH is co-founder, and shareholder in Surgical Information Sciences, Inc. VK has received grant support from Medtronic. LV is an editorial board member of Neurology and Therapy, and Brain Sciences. He has received consultant honoraria from Abbott, AbbVie Inc, and Boston Scientific, and research support from Medtronic, Boston Scientific, Abbott, AbbVie, Neuroderm, Biogen Inc. He has received NIH funding (R01 NS40902) as a site-PI. The remaining authors declare that the research was conducted in the absence of any commercial or financial relationships that could be construed as a potential conflict of interest.
